# Toward a more generalizable blood RNA signature for bacterial and viral infections

**DOI:** 10.1016/j.xcrm.2022.100866

**Published:** 2022-12-20

**Authors:** Rishi K. Gupta, Mahdad Noursadeghi

**Affiliations:** 1 Institute of Health Informatics, University College London, London, UK; 2 Division of Infection and Immunity, University College London, London, UK

## Abstract

Host-response profiles can discriminate different infections. A new 8-gene blood RNA signature to discriminate bacterial and viral infections extends our focus hitherto on the case mix from the US and Europe to include that of low- and middle-income countries.^1^ Challenges remain.

## Main text

Host response biomarkers of infectious disease that discriminate bacterial and viral infections have long been sought in clinical practice to guide antibacterial prescribing. Examples include measurement of circulating leukocytes, C-reactive protein (CRP), and pro-calcitonin supported by clinical trials that show these may reduce antibiotic prescriptions while achieving similar or even improved clinical outcomes.^2^^,^^3^^,^^4^

Much attention has focused on the application of blood transcriptional profiling combined with computational approaches to search multiparametric data for individual or combinations of measurements in pursuit of better biomarkers of bacterial and viral infections.^5^^,^^6^ Although this approach is based on the premise that the host response to these infections is distinct (as reflected in the blood transcriptome), computational biomarker discovery is generally agnostic of underlying biology. In fact, it purposefully jettisons data that do not add any discriminatory value to find the most parsimonious solution at the expense of our understanding of the biology. Instead, the most important consideration is the generalizability of the discovery dataset, encompassing heterogeneity of relevant pathogens, host biology and health care behaviors, or systems that may directly or indirectly influence the blood transcriptome at the time of sampling.

In this issue, Rao et al.^1^ draw attention to the paucity of representation of cases from low- and middle-income (LMIC) countries in discovery data used to derive previously published signatures. They argue that previous studies have failed to capture the diversity of microbial pathogens in LMIC settings, focusing particularly on a selection of bacteria that they group as intracellular pathogens. They collate a comprehensive repository of published data derived from the most geographically diverse settings available to date and curate a combined dataset using a state-of-the-art bioinformatic pipeline. They show that a selection of previously reported blood transcriptional signatures provide significantly lower accuracy for discriminating viral infections from nominal intracellular bacteria, over-represented in data from LMIC, compared to extracellular bacteria, over-represented in data from Europe and North America. To address this deficit, they derive a new 8-gene signature to give a bacterial or viral infection (BoVI) score and validate the performance of this score in two independent cohorts with microbiologically confirmed infections from Southeast Asia, where overall they achieve > 90% sensitivity and > 80% specificity for discriminating bacterial and non-bacterial infections. So, is this the final word in the field?

No doubt there will be technological and cost hurdles that will need to be resolved for clinical translation, but there are also important limitations in the current evidence. The impact of different immunocompromised states on host response biomarkers remains untested. It is notable that the BoVI8 signature may discriminate between tuberculosis and viral infections in merged datasets, since this has been an important limitation of previous blood transcriptional signatures of active tuberculosis.^7^ Nonetheless, the validation cohorts in the present study did not include any cases of tuberculosis, malaria, fungal, or protozoan infections. Moreover, how the BoVI8 score performs in bacterial and viral co-infection is not known, for example, in the context of acute respiratory virus disease. To address this eventuality, and the fact that people with suspected infections do not represent a binary (bacterial versus viral) diagnostic dilemma, the ability to ascribe a probability to the presence of multiple infection classes may be a better approach than simple two-class discrimination. In this case, multiple biomarkers predictive for each class of infection may need to be considered together. In a recent head-to-head comparison of another blood transcriptional signature and CRP to discriminate bacterial and viral infections, we found the transcriptional signature to be more useful in detection of viral infections, but CRP to be more useful in identifying bacterial infections.^8^

The current study also excludes participants without confirmed microbiological diagnoses—one might argue the very cases that need novel tests. The number of these indeterminate cases was not specified but can often represent a majority of people presenting with suspected infections. The rationale for their exclusion is that it is not possible to assess the performance of a new test if no gold-standard reference is available. However, the clinical utility of biomarkers to inform decision making is likely to be highly dependent on the classification of these indeterminate cases. We previously used an alternative blood transcriptional signature for differentiation of bacterial and viral infections to estimate the relative frequency of each class among indeterminate cases.^9^ Notably, this revealed cases predicted to be due to bacterial pathogens but resolved without antibacterial treatment, raising the possibility that bacterial infections can be self-limiting. Hence, biomarkers for bacterial infections used in isolation to trigger prescribing may have the unintended consequence of increasing unnecessary antibacterial use. Clinical translation will therefore require future interventional studies that test the downstream impact of biomarker stratification for specific interventions (e.g., antimicrobial treatment initiation) on meaningful outcomes, with benchmarking against existing host-response diagnostics, such as CRP and pro-calcitonin—which may well prove difficult to beat.

What test performance is required to empower clinical teams to withhold antibacterials? Anything but perfect sensitivity or negative predictive value is unlikely to impact prescriptions for patients with severe disease (e.g., severe sepsis) in which any substantial delay in antibacterial drugs significantly increases mortality.^10^ Hence, the real-world application of these biomarkers may be limited to non-severe illness to discourage early empirical antibacterial prescribing where there is a low probability of bacterial infection. Even with this restriction, biomarkers may substantially reduce antibacterial prescribing, particularly in primary care settings.^3^ While the motivation for the application of these biomarkers has generally focused on reducing antibacterial prescribing, there are also significant benefits to enhanced detection of viral infections by identifying cases for targeted pathogen-specific diagnostic testing and implementation of infection-control measures. The BoVI8 signature is a significant step forward because it shows proof in principle that extending training datasets leads to more generalizable solutions, but we expect further refinements to find the optimal blood transcriptional biomarkers for this application.
